# High Prevalence of Faecal Carriage of ESBL-Producing *Enterobacteriaceae* among Children in Dar es Salaam, Tanzania

**DOI:** 10.1371/journal.pone.0168024

**Published:** 2016-12-09

**Authors:** Marit G. Tellevik, Bjørn Blomberg, Øyvind Kommedal, Samuel Y. Maselle, Nina Langeland, Sabrina J. Moyo

**Affiliations:** 1 National Centre for Tropical Infectious Diseases, Department of Medicine, Haukeland University Hospital, Bergen, Norway; 2 Department of Clinical Science, University of Bergen, Bergen, Norway; 3 Department of Microbiology, Haukeland University Hospital, Bergen, Norway; 4 Department of Microbiology and Immunology, Muhimbili University of Health and Allied Sciences, Dar es Salaam, Tanzania; Ross University School of Veterinary Medicine, SAINT KITTS AND NEVIS

## Abstract

**Background:**

Faecal carriage of ESBL-producing bacteria is a potential risk for transmission and infection. Little is known about faecal carriage of antibiotic resistance in Tanzania. This study aimed to investigate the prevalence of faecal carriage of ESBL-producing *Enterobacteriaceae* and to identify risk factors for carriage among young children in Tanzania.

**Methodology/Principal Findings:**

From August 2010 to July 2011, children below 2 years of age were recruited in Dar es Salaam, including healthy community children (n = 250) and children hospitalized due to diarrhoea (n = 250) or other diseases (n = 103). ChromID ESBL agar and ChromID CARBA SMART agar were used for screening. Antimicrobial susceptibility testing was performed by the disk diffusion method. ESBL genotypes were identified by Real-Time PCR and sequencing.

The overall prevalence of ESBL carriage was 34.3% (207/ 603). The prevalence of ESBL carriage was significantly higher among hospitalized children (50.4%), compared to community children (11.6%; P < 0.001; OR = 7.75; 95% CI: 4.99–12.03). We found high prevalence of Multidrug-resistance (94%) among *Escherichia coli* and *Klebsiella pneumoniae* isolates. No resistance to carbapenems was detected. For the majority of isolates (94.7%) we detected a *bla*_CTX-M-15_-like gene. In addition, the plasmid mediated AmpC beta-lactamase CMY-2 was detected for the first time in Tanzania. ESBL prevalence was significantly higher among HIV positive (89.7%) than HIV negative (16.9%) children (P = 0.001; OR = 9.99; 95% CI: 2.52–39.57). Use of antibiotics during the past 14 days and age below 1 year was also associated with ESBL carriage.

**Conclusions/Significance:**

We report a high rate of faecal carriage of ESBL-producing *Enterobacteriaceae* among children below 2 years of age in Tanzania, particularly those with HIV-infection. Resistance to a majority of the available antimicrobials commonly used for children in Tanzania leaves few treatment options for infections when caused by these bacteria.

## Introduction

Antimicrobial resistance is a serious problem worldwide. Infections caused by resistant organisms pose an important challenge for treatment of both common and life-threatening infections. The World Health Organization (WHO) has declared infections caused by multidrug resistant bacteria as an emerging global health problem of major public health concern [1]. Beta-lactam antibiotics include penicillins, cephalosporins and carbapenems, and constitute the most important group of agents for combating bacterial infections. Extended Spectrum Beta-Lactamases (ESBLs) are enzymes capable of hydrolysing many beta-lactam antibiotics and thereby protect ESBL-producing bacteria from the action of these drugs. ESBL-producing bacteria are frequently associated with co-resistance to non-beta-lactam antimicrobial agents and resistance to several different antibiotics at the same time (Multidrug-resistance (MDR)), which may critically complicate the treatment of severe bacterial infections. High prevalence of ESBL-producing bacteria has been reported worldwide [1–3]. While there are a number of publications on ESBL-producing bacteria causing clinical infections [4–7], relatively few studies from the African continent report on carriage of ESBL-producing organisms [8–11].

While a better understanding of the impact on faecal carriage of ESBL-producing bacteria on subsequent development of infection is needed, carriage is a potential risk for transmission and infection [12–14], and of particularly concern in healthcare settings, especially in developing countries where infection control is often inadequate. Little is known about faecal carriage of ESBLs and antibiotic resistance in Tanzania, with only two studies which have been conducted in the northern part of the country [15, 16]. This study was conducted in the largest city of Tanzania, Dar es Salaam, with a population of about five million. We aimed to investigate the prevalence of faecal carriage of ESBL-producing *Enterobacteriaceae* and to identify risk factors for carriage among young children in Dar es Salaam, Tanzania.

## Materials and Methods

### Ethics statement

The study was approved by the Muhimbili University of Health and Allied Sciences Institutional Review Board in Dar es Salaam, Tanzania, by the Regional Committee for Medical and Health Research Ethics (REK) in Norway, and by the respective hospital authorities at the three study hospitals. Written informed consent was obtained from the parents or guardian on behalf of all the children enrolled in the study.

### Study population

The present work was part of a larger study assessing causes of diarrhoea among children. The study population and data collection have previously been described [17]. Briefly, this prospective study was performed between August 2010 and July 2011, in Dar es Salaam, the largest city in Tanzania with a population of more than four million. Children below 2 years of age were included and categorized into three different study groups; healthy community children attending child health clinics for immunization and growth monitoring with no history of diarrhoea for one month prior to the study enrollment (n = 250), children hospitalized due to diarrhoea (n = 250), and children admitted due to diseases other than diarrhoea, also with no history of diarrhoea during the last month (n = 103). The study hospitals were the three major hospitals in Dar es Salaam; Muhimbili National Hospital and Amana and Temeke Municipal district hospitals. A standardized questionnaire and patient files were used for collection of demographic and clinical information. Weight for age (WAZ), length for age (LAZ) and weight for length (WLZ) Z-scores were calculated using EPI Info (USD, Inc., Stone Mountain, GA). Children were categorized to have normal nutritional status, mild or severe malnutrition using Z-scores according to WHO criteria [18]. Use of antibiotics during the last 14 days prior to study enrollment was recorded.

### Sample material

One stool specimen from each community and hospitalized child was collected on inclusion in the study and cultured on MacConkey agar within six hours. For hospitalized children the sample was collected within the first 24 hours upon admission. A sweep of bacterial colonies from MacConkey agar was stored at—80°C. A portion of each specimen was shipped on dry-ice to Bergen, Norway, for further analysis, including ESBL screening and genotyping.

### Phenotypic screening for ESBL-producing *Enterobacteriaceae*

Frozen bacterial samples were sub-cultured on non-selective media and over-night cultures were re-suspended in 0.85% saline. 2–3 x 10^5^ CFU, resulting in abundant growth still enabling identification of single colonies with different morphology, was used as inoculum for screening. ChromID ESBL agar (for screening for ESBL-producing *Enterobacteriaceae*, both the classical ESBLs inhibited by clavulanic acid and the AmpC enzymes) and ChromID CARBA SMART (for combined screening for carbapenemase producing *Enterobacteriaceae* and the specific screening for OXA-48 producing *Enterobacteriaceae*), were used according to the manufacturer’s instructions (BioMérieux, Marcy l’Etoile, France). The following quality control strains were used: *Klebsiella pneumoniae* ATCC 700603, *Escherichia coli* ATCC 25922, and molecularly characterized strains harboring different combinations of beta-lactamase genes provided by the Norwegian National Advisory Unit on Detection of Antimicrobial Resistance including *E*. *coli* harboring *bla*_OXA-48_, *bla*_CTX-M-15_ and *bla*_CTX-M-14_, *E*. *coli* harboring *bla*_NDM-1_, *bla*_CMY-16_ variant, *bla*_CTX-M-15_, *bla*_OXA-1_, *bla*_OXA-10_ and *bla*_TEM-1_, *K*. *pneumoniae* harboring *bla*_OXA-48_ and *bla*_SHV-11_ and *K*. *pneumoniae* harboring *bla*_KPC-2_, *bla*_SHV-11_ and *bla*_TEM-1_.

### Identification and susceptibility testing of the bacterial isolates

Isolates were identified with MALDI-TOF MS using the Microflex LT instrument and MALDI Biotyper 3.1 software (Bruker Daltonics, Bremen, Germany). Only one isolate of each species was included for each patient. Antimicrobial susceptibility testing was performed by the disk diffusion method and classified as susceptible or resistant according to the European Committee on Antimicrobial Susceptibility Testing (EUCAST) guidelines for aztreonam, cefepime, cefoxitin, chloramphenicol, ciprofloxacin, gentamicin, meropenem, tigecycline and trimetoprim-sulfamethoxazole [19]. When there were no zone diameter breakpoints available for the disk concentration used (for cefotaxime, ceftazidime, doxycycline and piperacillin-tazobactam) or no zone diameter breakpoints yet available for an agent (fosfomycin), then the Clinical and Laboratory Standards Institute’s (CLSI) guidelines was used [20]. Isolates showing intermediate resistance were categorized as resistant. Confirmation of ESBL phenotype was performed using the BBL Sensi-Disc ESBL Confirmatory Test Disks (Becton Dickinson, Sparks, MD, USA). Quality control strains were included.

### Real-Time PCR and sequencing for detection and identification of ESBL genotypes

All isolates were examined for the presence of *bla*_CTX-M_ genes by a Real-Time PCR assay with forward primer CTXM-F 5’-ATGTGCAGYACCAGTAARGT-3’, and reverse primers CTXM-R1 5’-TGGGTGAAGTAAGTGACCAGA-3’ and CTXM-R2 5’-TGGGTAAARTAGGTCACCAGA-3’ (TIB Molbiol, Berlin, Germany), which target a 595 bp internal region present in all the five different CTX-M phylogenetic groups. Genomic DNA was extracted by a rapid boiling procedure and stored at -70°C until PCR analysis. The reaction mix included: 1 x SYBR Premix Ex Taq (Tli RNaseH Plus) (TaKaRa, Otsu, Japan), 0.4 μM each of the primers, 2 μl of sample DNA and water to a total volume of 25 μl. The Real-Time PCR assay was performed using a LightCycler 480 Instrument II (Roche Diagnostics, Mannheim, Germany), with cycling conditions as follows: 95°C for 30 sec, followed by 35 cycles at 95°C for 10 sec, 60°C for 10 sec and 72°C for 30 s each, and then cooled to 40°C for 30 s. All samples were run on LightCycler 480 Multiwell Plate 96, white (Roche), and sealed with LightCycler 480 Sealing Foil (Roche). Each run included duplicate of a positive control and multiple no-template controls. Amplicons were sequenced using the reverse primers and BigDye Terminator v1.1 Cycle Sequencing Kit (Applied Biosystems, Foster City, CA, USA) and an ABI PRISM 3730 DNA Analyzer (Applied Biosystems). Sequences were analyzed using the RipSeq software (Pathogenomix Inc., CA, USA).

Isolates with a negative CTX-M PCR result were examined for the presence of *bla*_SHV_ genes by a previous published method detecting SHV5/12-like ESBLs [21]. All isolates resistant to cefoxitin were examined for the presence of plasmid mediated AmpC beta-lactamase (*bla*_CMY-2_) as previously described [21].

### Statistical analysis

Statistical analysis was performed using SPSS Statistics version 23 (SPSS Inc., Chicago, IL, USA). Chi-square test was used to compare proportions. Univariate and multivariate analysis were performed using logistic regression. Multivariate analysis of characteristic features for faecal carriage of ESBL-producing *Enterobacteriaceae* included the following nine variables; sex, age, place of residence, parent level of education, underweight, stunting, wasting, HIV status and use of antibiotics. A P-value < 0.05 was considered statistically significant.

## Results

### Study population

Of the 603 children enrolled, 242 were females and 361 were males. Of these, 289 were from Ilala, 179 from Kinondoni and 135 from the Temeke district. Age distribution was 372 children equal to or below 12 months, and 231 children above 12 months. HIV testing results were available for 348 of the children, of whom 29 had a positive test result and 319 had a negative test result.

### Prevalence of screening positive ESBL-producing *Enterobacteriaceae*

Of all 603 children, 34.3% screened positive for ESBL by the ChromID ESBL agar. None of the samples screened positive for carbapenemases using the ChromID CARBA SMART media. The prevalence of ESBL carriage for the different study groups is shown in Table 1. ESBL prevalence was significantly higher among both children hospitalized due to diarrhoea and among children hospitalized due to diseases other than diarrhoea, than among community children. The difference between the two groups with hospitalized children was not statistically significant.

**Table 1:** Prevalence of ESBL screening positive children in the different study groups.

Study group	ESBL screening positive (%)	P; OR (95% CI)
Community children	29 (11.6)	1
Children hospitalized due to diarrhoea	118 (47.2)	< 0.001; 6.81 (4.30–10.79)
Children hospitalized due to other diseases	60 (58.3)	< 0.001; 10.63 (6.13–18.44)

### Identification and susceptibility testing of the bacterial isolates

Screening identified 284 bacterial isolates from 207 children. Of these, 139 were identified as *K*. *pneumoniae*, 129 were *E*. *coli*, 11 were *Enterobacter cloacae* complex, 2 were *Klebsiella oxytoca*, 2 were *Citrobacter* spp. and 1 was *Proteus mirabilis*. Carriage of two different bacteria spp. was detected in 75 (36.2%) of the participants with a positive screening. Only one sample contained more than two different bacteria spp. The prevalence of more than one ESBL-producing isolate was highest among the screening positive children hospitalized due to diarrhoea (56; 47.5%), followed by the screening positive children hospitalized due to other diseases (16; 26.7%) and the screening positive community children (4; 13.8%).

Resistance to different antimicrobial agents is shown in Table 2. The ESBL screening positive isolates showed high rates of resistance to antimicrobials commonly used in Tanzania. All isolates were susceptible to meropenem. MDR, defined as resistance to three or more categories of antimicrobial agents other than cephalosporins and aztreonam, was highly prevalent, with around 94% of *E*. *coli* and *K*. *pneumoniae* and more than 80% of *E*. *cloacae* complex isolates being MDR. Co-resistance to ciprofloxacin, gentamicin and trimethoprim-sulfamethoxazole was detected in about half of the *E*. *coli* and *E*. *cloacae* complex isolates and in about 14% of the *K*. *pneumoniae* isolates.

**Table 2:** Susceptibility to antimicrobial agents for the ESBL screening positive bacteria.

	Prevalence of resistant isolates (%)					
	*E*. *coli*	*K*. *pneumoniae*	*K*. *oxytoca*	*E*. *cloacae* complex	*P*. *mirabilis*	*Citrobacter* spp.
Antimicrobial agent	(N = 129)	(N = 139)	(N = 2)	(N = 11)	(N = 1)	(N = 2)
Aztreonam^1^	97.7	99.3	100	100	0	50
Cefepime^1^	97.7	98.6	100	90.9	0	50
Cefotaxime^2^	99.2	100	100	100	0	50
Cefoxitin^1^	15.5	1.4	0	100	0	0
Ceftazidime^2^	96.9	97.8	100	100	0	50
Chloramphenicol^1^	41.9	71.2	100	81.8	100	0
Ciprofloxacin^1^	62.8	17.3	100	63.6	100	0
Doxycycline^2^	80.6	25.2	0	54.5	100	0
Fosfomycin^2^^,^^3^	0	1.4	0	0	0	0
Gentamicin^1^	77.5	92.8	100	90.9	0	50
Meropenem^1^	0	0	0	0	0	0
Piperacillin-Tazobactam^2^	45.7	84.2	100	36.4	0	50
Tigecycline^1^^,^^3^	0	7.2	0	0	0	0
Trimethoprim-Sulfamethoxazole^1^	98.4	99.3	100	81.8	100	0
Multidrug-resistance I^4^	93.8	94.2	100	81.8	100	0
Multidrug-resistance II^5^	50.4	14.4	100	63.6	0	0

^1^EUCAST guidelines applied.

^2^CLSI guidelines applied.

^3^Zone diameter breakpoints validated for *E*. *coli* only.

^4^Multidrug-resistance I defined as resistance to three or more of the antibacterial agents in the table.

^5^Multidrug-resistance II defined as resistance to ciprofloxacin, gentamicin and trimethoprim-sulfamethoxazole.

### ESBL genotypes

A CTX-M genotype was detected in 94.7% (269/ 284) of the ESBL screening positive bacteria from 198 children. The distribution of a CTX-M-15 like genotype among the different bacteria spp. was as follows: 121 *E*. *coli*, 133 *K*. *pneumoniae*, 2 *K*. *oxytoca*, 10 *E*. *cloacae* complex and 1 *Citrobacter* spp. A CTX-M-14 like genotype was found in 2 *E*. *coli*. An ESBL type *bla*_SHV_ gene, SHV-5/12 like, was detected in 4 of the CTX-M negative isolates. Three of the 33 isolates resistant to cefoxitin were positive for CMY-2. They were all *E*. *coli* and two of them were also harboring CTX-M-15 like genes.

### Concordance between ESBL screening, genotypes and phenotypes

Of all the 284 screening positive isolates, 9 of them were negative using the ESBL confirmatory test disks. Of these, 3 isolates were CMY-2 positive, 3 isolates were resistant to cephalosporins, and the last 3 isolates were sensitive to cephalosporins. Only for five of the ESBL screening positive patients an ESBL genotype was not found.

### Characteristics of faecal carriage of ESBL-producing *Enterobacteriaceae*

**Fig 1:**
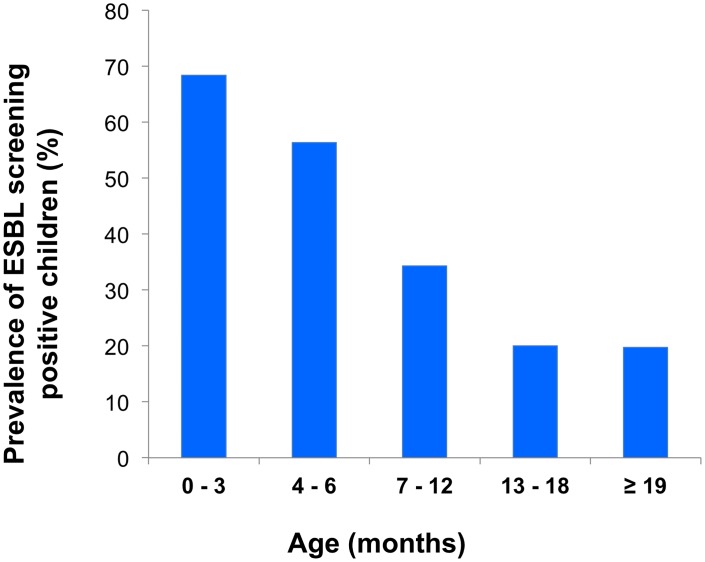
Prevalence of ESBL carriage in different age groups. The graph shows the prevalence (%) of ESBL carriage among the study participants when categorized into five different age groups.

The characteristics of faecal carriage of ESBL-producing isolates are shown in Table 3. HIV status was not known for all children, therefore multivariate analysis was first performed by including all children but without HIV status as a variable, and then by only including the 348 children with known HIV status. Multivariate analysis showed that age equal to or below 12 months was significantly associated with ESBL carriage (P = 0.012; OR = 1.82; 95% CI: 1.14–2.91). To further assess the impact of age, children were categorized into five age groups. The prevalence of ESBL carriage in these five age groups is illustrated in Fig 1. With a prevalence of 68.4% (39/57) among children aged 0–3 months (P < 0.001; OR = 8.83; 95% CI: 3.87–20.15, of 56.3% (36/64) among children aged 4–6 months (P < 0.001; OR = 5.24: 95% CI: 2.40–11.16) and 34.3% (86/251) among children aged 7–12 months (P = 0.025; OR = 2.13; 95% CI: 1.10–4.11), infants had a significantly higher risk of carrying ESBL than children above 12 months.

**Table 3:** Characteristics of faecal carriage of ESBL screening positive bacteria among children in Dar es Salaam, Tanzania.

Characteristics		ESBL screening positive children			
	NChildren	n (%)	Univariate	Multivariate^2^	Multivariate^3^
			P; OR (95% CI)	P; OR (95% CI)	P; OR (95% CI)
**Sex**					
Male	361	134 (37.1)	0.078;1.37 (0.97–1.94)	0.24; 1.27 (0.85–1.91)	0.58; 1.20 (0.63–2.31)
Female	242	73 (30.2)	1	1	1
**Age**					
≤ 12 months	372	161 (43.3)	<0.001; 3.07 (2.09–4.50)	0.012; 1.82 (1.14–2.91)	0.55; 1.25 (0.61–2.54)
**>** 12 months	231	46 (19.9)	1	1	1
**Place of residence (district)**					
Ilala	289	89 (30.8)	1	1	1
Temeke	135	44 (32.6)	0.710; 1.09 (0.70–1.68)	0.91; 1.03 (0.62–1.70)	0.40; 1.55 (0.56–4.29)
Kinondoni	179	74 (41.3)	0.020; 1.58 (1.07–2.34)	0.001; 2.62 (1.49–4.60)	0.014; 3.34 (1.28–8.72)
**Parent level of education**					
Higher level	9	7 (77.8)	0.017; 6.90 (1.42–33.50)	0.34; 2.19 (0.43–11.16)	P = 1.0
Secondary, Primary and below	594	200 (33.7)	1	1	1
**Children groups**					
Community	250	29 (11.6)	1	1	1
Diarrhoea	250	118 (47.2)	<0.001; 6.81 (4.30–10.79)	<0.001; 5.32 (3.13–9.04)	<0.001; 5.98 (2.28–17.72)
Other diseases	103	60 (58.3)	<0.001; 10.63 (6.13–18.44)	<0.001; 10.08 (5.39–18.83)	<0.001; 34.32 (2.78–424.14)
**Nutritional status**					
**WAZ**					
Normal weight	329	104 (31.6)	1	1	1
Underweight	274	103 (37.6)	0.12; 1.30 (0.93–1.83)	0.15; 1.42 (0.89–2.29)	0.99; 1.00 (0.48–2.09)
**LAZ**					
Normal	257	68 (26.5)	1	1	1
Stunted	346	139 (40.2)	<0.001; 1.87 (1.31–2.65)	0.12; 1.45 (0.91–2.31)	0.20; 1.65 (0.76–3.56)
**WLZ**					
Normal	454	163 (35.9)	1	1	1
Wasting	149	44 (29.5)	0.16; 0.75 (0.50–1.12)	0.48; 0.83 (0.48–1.41)	0.15; 0.50 (0.20–1.29)
**HIV**					
Positive	29	26 (89.7)	<0.001; 42.53 (12.43–145.57)	Not applicable	0.001; 9.99 (2.52–39.57)
Negative	319	55 (16.9)	1		1
**Use of antibiotics**^1^					
No use	378	100 (26.5)	1	1	1
Used	225	107 (47.6)	<0.001; 2.52 (1.78–3.57)	0.022; 1.61 (1.07–2.41)	0.37; 1.38 (0.68–2.80)

N: Total number of samples tested; n: number of positive samples; WAZ: Weight-for-age-Z-score; LAZ: Length-for-age-Z-score; WLZ: Weight-for-length-Z-score

^1^Use of antibiotics during the last 14 days prior to inclusion, or on inclusion.

^2^ and ^3^ Multivariate analysis for all children (n = 603) and only children with known HIV status (n = 348), respectively.

Living in the Kinondoni district was significantly associated with ESBL carriage in both univariate and multivariate analysis (P = 0.020; OR = 1.58; 95% CI: 1.07–2.34 (univariate) and P = 0.001; OR = 2.62; 95% CI: 1.49–4.60 (multivariate)). Further, in both univariate and multivariate analysis, ESBL carriage was significantly more prevalent in HIV positive children than in HIV negative children (P < 0.001; OR = 42.53; 95% CI: 12.43–145.57 (univariate) and P = 0.001; OR = 9.99; 95% CI: 2.52–39.57 (multivariate)). Stunting was significantly associated with ESBL carriage in the univariate analysis including all children (), but was not a significant risk factor in the multivariate analysis.

Children who had used antibiotics during the last 14 days prior to study enrollment were more likely to carry ESBL-producing strains that those who had not taken antibiotics (P = 0.022; OR = 1.61; 95% CI: 1.07–2.41).

In univariate analysis, children of parents with a higher level of education were significantly more likely to be ESBL carriers than those with lower levels of education (P = 0.017; OR = 6.90; 95% CI: 1.42–33.50), but this association was not significant in multivariate analysis.

The prevalence of ESBL carriage upon admission at the different study hospitals was also assessed. The prevalence of ESBL carriage upon admission was significantly higher among children admitted to Muhimbili National Hospital (76%, 98/129) than among those admitted to Amana District Hospital (39.2%, 65/166) or Temeke District Hospital (25.9%, 15/58, P < 0.001; OR = 9.06; 95% CI: 4.44–18.59) (Table 4).

**Table 4:** Prevalence of ESBL carriage among children admitted at each of the study hospitals.

Hospital	ESBL screening positive (%)	P; OR (95% CI)
Muhimbili National Hospital	98/129 (76)	< 0.001; 9.06 (4.44–18.59)
Amana District Hospital	65/166 (39.2)	0.07; 1.85 (0.95–3.59)
Temeke District Hospital	15/58 (25.9)	1

## Discussion

This is the first study from Tanzania reporting on ESBL carriage among very young children, including both healthy and sick children from the community. Faecal carriage of ESBL producing *Enterobacteriaceae* has been documented in both children and in adults [8, 10, 15, 22–24]. A global prevalence of 14% ESBL carriage among healthy individuals has been reported [23].

Data on ESBL carriage among community children on the sub-Saharan continent are limited [9, 11, 15, 25], and numbers of children included in previous studies are low. There is only one publication on ESBL carriage in the community in Tanzania [15]. The overall prevalence of ESBL carriage is comparable to the prevalence of about 30% found among children admitted at hospitals in Guinea-Bissau, Gabon and Niger [8, 9, 26]. The ESBL carriage differed significantly between healthy and hospitalized children. Carriage rate among healthy community children in our study is comparable to the prevalence of 16.5% found in community settings in Mwanza in northern Tanzania, including both children and adults [15]. However, it is twice the prevalence found among healthy children in France [27], but still half the prevalence found among children in Laos and Lebanon [22, 28] and much lower than the prevalence of 59% found among children in Bangui [11]. We observed a very high carriage rate among hospitalized children. This is higher than in studies from Guinea-Bissau, Gabon, Madagascar and Niger [8, 9, 25, 26]. At the hospital level, alarmingly 76% of the children are ESBL carriers when admitted at the Muhimbili National Hospital. Data on previous hospitalization was not available, but as this is a national referral hospital, it is likely that a substantial proportion of these children have a history of previous hospitalization, which might have contributed to colonization. This is supported by studies showing acquisition rates during hospitalization of 47.5% and 94%, respectively [25, 26].

We found carriage of more than one bacterial species more common among children admitted due to diarrhoea. Carriage of more than one bacterial species might increase the risk of transfer of genetic elements carrying resistance genes. In this study we did not aim for extensive molecular characterization, however the high prevalence of the CTX-M-15 like genotype further confirms the CTX-Ms as the dominant enzyme among carriers both in Tanzania and worldwide [15, 23]. The plasmid mediated AmpC gene *bla*_CMY-2_ was detected in three *E*. *coli* isolates, and this is the first report of CMY-2 in Tanzania. Only *bla*_CTX-M_ negative isolates were assessed for presence of *bla*_SHV_, hence the prevalence of SHV-type ESBLs might be underestimated. With an ESBL genotype identified for 97.6% of the patients, the screening media showed high specificity, which possibly would have been higher if other ESBL genotypes were search for.

The ESBL positive isolates showed high rates of resistance to antimicrobials available and commonly used in Tanzania, and very high levels of MDR. Empirical treatment according to guidelines in this setting has been associated with high case-fatality rates [29]. Resistance to the majority of drugs most commonly used for infections in children leaves very few choices for treatment. WHO recommend continued co-trimoxazole prophylaxis until adulthood for all HIV positive children in settings like Tanzania. Considering a resistance rate for co-trimoxazole close to 100%, together with a high level of co-resistance to other commonly used antimicrobials, adhering to these guidelines might contribute to increased selection for resistance against other available antimicrobial agents. Carbapenemases were not detected, but the study was conducted around the time at which carbapenems were introduced in Tanzania, hence new studies on the prevalence of these enzymes are needed.

We identified younger age, HIV infection and use of antibiotics as independent factors associated with ESBL carriage. Notably, being a resident of the Kinondoni district, which settles people with a higher level of income, was also significantly associated with ESBL carriage. In a setting were economical resources are limited, higher income increases affordability to antimicrobial treatment. Living in a highest income family was the only risk factor for carriage in the study from Bangui [11]. Several studies have reported on age as a risk factor for carriage, but the impact of age on faecal ESBL carriage varies with the age range of the study population and the study setting [9, 15, 23, 30]. Although our study included young children within a limited age range, younger age was significantly associated with ESBL carriage. Both the very youngest children, aged 0–3 months, and those aged 4–6 months, were nine and five times more likely to be colonized with ESBL positive bacteria than the oldest children, respectively. This may reflect health-care associated transmission or resistant strains being transmitted from mothers to their babies, possibly during delivery. Interestingly, Nelson et al. did not find any phenotypic similarity between ESBL strains from women and their newborns, and half of the newborns acquired ESBL producing bacteria already at their first day of life [16]. In contrast, Denkel et al. did identify ESBL colonization of the mother as an independent risk factor for colonization of neonates [31]. However, results should be interpreted with caution, as risk factors might not be the same in a high prevalence setting in the developing world as in a low prevalence setting in the developed world.

HIV positive children were ten times more likely to be ESBL carriers than those who were HIV negative. HIV status has not been well documented as a risk factor for ESBL carriage. HIV positive children are more prone to infections and hence more likely to be hospitalized and consume antimicrobials than HIV negative children. HIV-infection has been associated with resistant bacteria [32–34]. Considering the very strong association between ESBL carriage and HIV-infection, together with a positive HIV status of 8% among these very young children, further elucidation is urgent.

Stunted children were significantly more at risk of ESBL carriage. Malnourished children with impaired immunity are more vulnerable to infections, and are hence more likely to be treated with antibiotics. Previous use of antibiotics as far back as 12 months before sampling has been described as a risk factor for carriage [22, 23]. Even though only very recent data on use of antimicrobials were available, use of antimicrobials the last 2 weeks prior to study enrollment was significantly associated with ESBL carriage. Children that used antibiotics had nearly a two-fold increased risk for carriage compared to those that did not use antibiotics. If a longer history of use was available, this link could possibly be even stronger.

Immediate action is needed to prevent resistant bacteria spreading in the community and healthcare facilities. In developing countries, high level of antibiotic consumption is related to high prevalence of infections. To reduce the use of antibiotics, stewardship and guidance on appropriate use is extremely important. Prevalence of infections can be reduced by better access to and coverage of vaccination, better hygienic precautions, improving sanitation, and improvement of nutritional status. Retroviral treatment of HIV positive pregnant women to reduce mother-to-child transmission should be increased.

This study highlights a worryingly high faecal carriage of ESBL producing—and MDR bacteria among children below 2 years of age. It is the first study on ESBL carriage among young community children in Tanzania, including both healthy children and children with illness. Few studies exist from other sub-Saharan countries. Treatment options for infections caused by these bacteria are limited. The link between carriage and infection needs further elucidation. Carriage contributes to a considerable circulating pool of resistance genes and strict antibiotic stewardship and measures to prevent infections need immediate attention.
